# Liver Is T Cells' Ace in the Hole

**DOI:** 10.1371/journal.pbio.1000113

**Published:** 2009-05-26

**Authors:** Mary Hoff

**Affiliations:** Freelance Science Writer, Stillwater, Minnesota, United States of America

The intricacies of the adaptive immune system are a wonder to behold. Perceived invaders bearing surface characteristics, known as antigens, are picked up by ever-vigilant, widely circulating antigen-presenting cells (APCs). APCs carry the antigens to lymph nodes and other secondary lymphoid tissues (SLTs), where they are used as templates for mobilizing large numbers of lymphocytes—T cells and B cells—that have receptors designed to recognize that particular antigen.[Fig pbio-1000113-g001]

**Figure pbio-1000113-g001:**
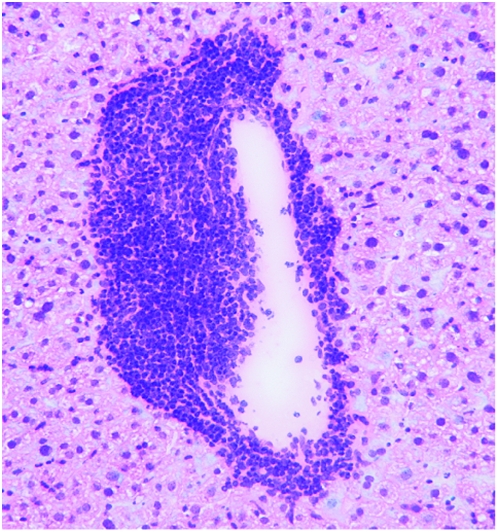
A remnant from the time before lymph nodes evolved: These lymphoid aggregates in the adult liver can prime cell-mediated immunity and provide a niche for T cell–antigen encounters.

It's long been thought that this kind of immune response depends on the ability of APCs and lymphocytes to meet in the SLT. Indeed, specific areas within the lymph nodes called germinal centers demarcate where B cells divide in response to the presence of an antigen, creating high-affinity antibodies to flood the body and battle the invader. Yet cold-blooded vertebrates, which lack lymph nodes, are still able to respond robustly to the introduction of antigens by T cell proliferation. Is there a mystery participant in the immune response that might provide an alternate venue where lymphocytes and antigen-bearing APCs might interact and initiate the adaptive immune responses in the absence of SLTs? Using strategic combinations of various mutant mice, disease-mimicking injections, and artful experiments, Melanie Greter, Janin Hofmann, and Burkhard Becher concluded that in the case of T cells, the liver plays just such a role.

The team started by challenging mice that were engineered to lack any secondary lymphoid tissues (*aly/aly* mice) with experimental autoimmune encephalomyelitis (EAE), a model for multiple sclerosis that does not require a B cell response and so could be used to home in on T cell action. As expected, the antigen didn't induce autoimmune disease in the mutants, though it did in controls. Further, when cells reactive to the antigen were harvested from *aly/aly* mice and expanded, they were unable to induce an immune response in either mutant or control animals. However, when *aly/aly* mice were supplied with T cells specific for the antigen, they did indeed expand them, showing that T cells can answer the call to action even when SLTs are not present.

The problem with *aly/aly* mice could be one of two things: lack of adequate meeting places (that is, SLTs) for APCs and T cells, or dysfunctional immune cells. To test which was the case, the researchers used irradiation and bone marrow transplant to make two kinds of chimeric mice: ones with SLTs but with *aly/aly* immune system cells, and one deficient in lymph nodes but with normal immune cells. Surprisingly, they discovered that the latter responded normally to EAE, while the former remained unsusceptible, even though SLTs were intact. Additional tests supported the conclusion that the *aly/aly* problem is not due to structural defects in the lymphatic system itself but to defects in immune system cells.

The researchers wondered whether the spleen, the last remnant of SLT in *aly/aly* mice, might be filling in for the missing lymph nodes in the *aly/aly* mice with normal immune cells. To test this, they splenectomized the mice before immunization. A clear immune response to immunization confirmed that the spleen was not responsible for the ability of these mice to launch a T cell–powered response to antigen.

Can B cells also be activated in the absence of SLTs? To test that, the researchers injected the antigen KLH, which initiates a delayed hypersensitivity response after immunization, into the earskin of *aly/aly* and control mice. Both showed ear swelling, an indication of cell-based immune response, but neither *aly/aly* mice nor *aly/aly* mice with transplanted immune systems (with or without spleens) were able to launch a humoral immune response. The researchers concluded that SLTs are necessary for B cell but not T cell activation.

The fact that T cells are able to respond to antigen in the absence of SLTs raises the question of where priming, normally an SLT function, occurs. By injecting the various immune system chimeras with different fluorescent tracers in different combinations and locations, the researchers discovered that in chimeras without SLTs, antigens are transported by APCs into the liver. Histological studies subsequently showed that neo-lymphoid aggregates of T cells and APCs had been induced in the periportal areas of the liver.

The researchers went on to demonstrate that the liver, as helpful as it is for T cell–mediated immune response, doesn't provide B cells the support they need to do their job. Is this T cell promiscuity/flexibility restricted to helper T cells, or can killer T cells also be activated outside of SLTs? Using a mouse melanoma model, the authors also proved that SLT-less mice are able to develop a killer T cell–based response against tumor cells. Additional analysis of mice with intact SLTs but a mutation that inhibits T cells from entering them showed that such neo-lymphoid tissue is induced in the liver with immunization even when SLTs are present but inaccessible.

This study shows that structural requirements for T cell and B cell responses differ markedly, with the liver providing a back-up venue for the T cell–mediated immune response in the absence of SLTs. The authors note that their findings are consistent not only with observations of immune function in SLT-less cold-blooded vertebrates, but also with the fact that the livers of mammalian fetuses function as lymphoid organs, and that food allergies can be acquired through liver transplantation even in the adult.

This elegant demonstration of distinct systems for activation of B cells and T cells and for a backup function of the liver provides valuable insights into the intricacies of immunity. Though further research is needed to elucidate the details of T cells' liver-based ability to initiate an adaptive immune response as well as the complexities of B cell priming, this study takes a giant step toward better understanding of the mammalian immune system, and thus the development of improved therapies for infectious diseases, cancer, and autoimmunity.

**Greter M, Hofmann J, Becher B (2009) Neo-Lymphoid Aggregates in the Adult Liver Can Initiate Potent Cell-Mediated Immunity. doi:10.1371/journal.pbio.1000109**

